# Effect of the Replacement of Corn (*Zea mays*) by Chickpea (*Cicer arietinum*) and Rice (*Oryza sativa*) in Extruded Snacks on Protein Digestibility, Mineral Bioaccessibility and Estimated Glycemic Index

**DOI:** 10.3390/life16081322

**Published:** 2026-08-12

**Authors:** Antonio L. García-Cordero, Ehira Romero-Castelán, Juana Fernández-López, Fernando Díaz-Sánchez, Jose M. Lorenzo, Jose A. Rodriguez, Raquel Lucas-González, Eva M. Santos

**Affiliations:** 1 Área Académica de Química, Universidad Autónoma del Estado de Hidalgo, Ctra. Pachuca-Tulancingo Km 4.5 s/n, Col. Carboneras, Mineral de la Reforma 42183, Hidalgo, Mexico; aluisgarciacordero@gmail.com (A.L.G.-C.); ehira_romero@uaeh.edu.mx (E.R.-C.); josear@uaeh.edu.mx (J.A.R.); 2 IPOA Research Group, Institute for Agri-Food and Agro-Environmental Research and Innovation, Miguel Hernández University (CIAGRO-UMH), 03312 Orihuela, Alicante, Spain; j.fernandez@umh.es; 3 Fritos TOTIS, C/Sur 4, 123, Tizayuca 43800, Hidalgo, Mexico; fdiaz@totis.com.mx; 4 Centro Tecnológico de la Carne de Galicia, Avda. Galicia nº 4, Parque Tecnológico de Galicia, 32900 San Cibrao das Viñas, Ourense, Spain; jmlorenzo@ceteca.net; 5 Área de Tecnoloxía dos Alimentos, Facultade de Ciencias, Universidade de Vigo, 32004 Ourense, Ourense, Spain

**Keywords:** pulses, healthy snacks, extrusion, digestibility, mineral bioaccessibility, phytic acid

## Abstract

This study evaluated the effect of chickpea and rice incorporation to replace corn in extruded snacks on protein digestibility, mineral bioaccessibility, and estimated glycemic index (eGI) using a standardized in vitro digestion model (INFOGEST 2.0). Four formulations were analyzed: a control (100% corn, CF) and chickpea-enriched snacks (F30, F60, and the commercial flavored F60 formulation, CCP). Chickpea replacement increased protein, dietary fiber, and mineral contents while reducing total starch. Protein digestibility remained high across all samples (89.5–96.6%), although slightly lower in chickpea/rice-enriched formulations. However, these formulations provided a higher amount of digestible protein because of their increased protein content. Mineral bioaccessibility varied depending on the element, but chickpea and rice incorporation generally enhanced mineral concentrations in the fraction potentially available for absorption despite the presence of phytic acid and insoluble dietary fiber. Starch hydrolysis was rapid during the early stages of digestion, leading to high estimated glycemic index (eGI) values in all samples. Nevertheless, chickpea/rice-enriched formulations exhibited lower eGI values compared to the corn control, likely due to reduced starch content and matrix effects. Overall, chickpea and rice replacement improved the nutritional profile of extruded snacks by enhancing protein quality and mineral availability, despite its impact on glycemic response being limited, highlighting opportunities for further product optimization.

## 1. Introduction

The consumption of small food portions between main meals to suppress hunger is commonly referred to as snacking [1,2]. Snack consumption is frequently associated with refined fats and energy-dense, nutrient-poor, and high-sodium components—components known to exert detrimental health effects [2]. Accordingly, the regular consumption of traditional snack foods has been linked to adverse health outcomes and an increased risk of disease development (hypertension, diabetes, cancer and cardiovascular diseases) [3,4,5].

In the case of expanded extruded snacks, these are traditionally produced from refined cereal flours, such as corn and rice, and are predominantly starch-based [6], exhibiting virtually no inherent flavor or aroma. After extrusion and expansion, a post-extrusion flavored oil coating is typically applied, which may account for approximately one-fifth to one-third of the final product mass, serving as the main carrier for flavoring and coloring agents that confer the characteristic aroma and appearance to the product but also increase the presence of fat, negatively affecting the nutritional profile of those products [7,8]. In this sense, extruded snacks have a wide margin for improvement, reducing those unhealthy aspects due to their composition.

The primary strategy employed by the food industry to enhance the nutritional profile of these foods has been the incorporation of additional ingredients that enhance the nutritional profile, such as pulses, vegetables, or industrial by-products [9]. Pulses provide a higher protein content, increasing the content of dietary fiber and minerals, while having a lower contribution of carbohydrates compared to traditional ingredients such as cereals [10,11,12,13,14,15,16]. The presence of plant-based proteins and insoluble dietary fiber has been associated with reduced systemic inflammation, improved insulin sensitivity, and a lower risk of developing type 2 diabetes mellitus [17]. Moreover, the combination of cereals and legumes may help to improve the balance of essential amino acids, thereby enhancing protein quality [18]. Among these pulses, chickpea is of particular interest due to its high availability and colorless material. Compared with corn, chickpea contains substantially higher levels of protein (19–27% vs. 8–15%) and total dietary fiber (6–25% vs. 4–14%), with insoluble fiber representing the predominant fraction in both raw materials (10–18% in chickpea and 4–5% in maize) [19,20,21]. Chickpea proteins are also rich in lysine, a deficient amino acid in corn, contributing to a more balanced amino acid profile [22]. In addition, chickpea can also provide relevant minerals comparing to corn, including calcium (82–272 vs. 7–8 mg/100 g), iron (4.6–7.5 vs. 1–3 mg/100 g), magnesium (147–195 vs. 100–127 mg/100 g), potassium (994–1264 vs. 143–287 mg/100 g), phosphorus (394–452 vs. 210–400 mg/100 g), and zinc (3.4–4.4 vs. 1–2 mg/100 g) [23,24].

Several works using chickpea in extruded snack formulations have been published, focused mainly on the physicochemical, technological, and sensorial parameters [1,11,16,25,26,27].

However, these beneficial nutritional effects of incorporating chickpea in snacks can be overshadowed by texture challenges since snack expansion, hardness, and color are affected [18]. In this regard, the addition of rice to pulse formulations contributes to lightening the darker color of chickpea and, above all, mitigating the hardness, increasing expansion rates [28,29].

On the other hand, while the increase in dietary fiber in snacks could have a positive impact on starch hydrolysis, decreasing the glycemic index, as it has been reported in previous in vivo studies conducted with low-carbohydrate and high-fiber diets [30,31,32,33,34], the digestibility of proteins and mineral bioaccessibility can also be altered. The presence of insoluble dietary fiber and antinutritional compounds such as phytic acid have been associated with reduced mineral absorption and bioaccessibility [35,36]. Phytic acid exerts a strong chelating effect on minerals such as Ca, Cu, Fe, Mg, Mn, P, and Zn, limiting their release during digestion and subsequent absorption [37,38,39,40], even though the phytic contents may decrease during the extrusion process [16,39,41]. Although Neder-Suarez et al. [27] investigated in vitro protein digestibility of chickpea–rice snacks, studies evaluating mineral bioaccessibility in snacks are very limited [42], and, to the best of our knowledge, no such studies have been reported for commercial snack products.

In this context, understanding the behavior of foods during the digestion process is essential to link diet to health benefits, as it allows the analysis of physiological responses to specific foods. Several models have been developed to simulate human gastrointestinal digestion. To investigate food–human interactions, in vivo and in vitro models have been proposed. In vivo approaches are considered the most physiologically realistic. However, they are also the most invasive and costly and raise significant ethical concerns [43]. This category also includes the use of laboratory animals, which involves surgical placement of cannulas in the digestive tract for sample collection, procedures that often result in the death of the animal. Moreover, it is important to note that the similarity between human and animal gastrointestinal processes remains debatable [44].

In contrast, in vitro digestion models, which were applied in the present study, offer several advantages, including experimental simplicity, ease of distinguishing the different phases of the digestive process, good inter-laboratory reproducibility, robustness, and lower costs compared with in vivo models. Nevertheless, these systems could present some limitations, as they cannot fully replicate the dynamic complexity of digestion or the physiological interactions with the organism [44]. Despite their inherent limitations, standardized and harmonized protocols such as INFOGEST 2.0 have emerged as robust tools that improve the consistency and comparability of experimental results, with demonstrated agreement with in vivo data [44,45,46,47]. Moreover, these models have been widely validated for the evaluation of bioaccessibility in diverse food systems and for investigating critical factors that govern the digestion and bioaccessibility of various food components and matrices [48]. Consequently, incorporating protein- and fiber-rich ingredients into snacks offers a promising, healthier alternative to cereal combinations, potentially lowering the glycemic index and improving the essential amino acid profile.

Based on the current evidence, the hypothesis of this study is that partial replacement of corn with chickpea and rice improves the nutritional functionality of extruded snacks by enhancing protein quality and mineral bioaccessibility while reducing starch digestibility and the estimated glycemic index after gastrointestinal digestion, without markedly compromising protein digestibility. Therefore, this study aimed to evaluate the effect of replacing corn with chickpea and rice in extruded corn snacks on protein digestibility, estimated glycemic index, and mineral bioaccessibility following in vitro gastrointestinal digestion in the development of a healthier snack.

## 2. Materials and Methods

### 2.1. Snack Elaboration

Extrusion processing was carried out at the industrial facility of Fritos Totis (Tizayuca, Hidalgo, Mexico) using a twin-screw extruder (Evolum, Clextral, Firminy, France). The feed moisture content ranged from 11 to 16%. The operating conditions were as follows: feed rate 200–425 kg/h, screw speed 550–649 rpm, screw temperature 173–177 °C, barrel temperature 28–145 °C, and die pressure 45–60 bar. Water was injected into the barrel at 23–25 L/h, resulting in a specific mechanical energy (SME) of 100–126 Wh/kg. Extrudates were obtained through a die with a diameter of 4.6 mm.

Based on preliminary experiments, four formulations were studied (Table 1). The formulations were chosen considering a low and a high proportion of chickpea, and an adequate combination of rice to offer a product with a good texture and appearance according to the standards of the company (Fritos Totis, Mexico). The formulations included a control sample (100% corn, CF) and chickpea–rice-enriched formulations containing 30/50% (F30) and 60/35% (F60) chickpea/rice flour, respectively. These three samples were produced without flavored oil coating. An additional formulation corresponding to a commercial product derived from the F60 formulation but with the application of a flavored oil coating, CCP (Fritos Totis, Mexico), with an approximate final chickpea content of 45%, was also evaluated. This formulation was included in the evaluation to have better information about the behavior of the final snack, which is usually sold with flavoring. In this case, the flavoring corresponded to cheddar cheese (milk solids, starch, maltodextrin, iodized salt, sugar, glucose, natural and nature-identical flavorings, citric acid, silicon dioxide, modified starch, calcium silicate, sodium caseinate, lactic acid, calcium lactate, mono- and diglycerides of fatty acids, disodium phosphate, sodium citrate, xanthan gum, triacetin, and propylene glycol) with palm olein as the oil vehicle (Fritos Totis, Mexico). Nevertheless, limitations of comparing CCP with the experimental formulations (CF, F30, and F60) have to be highlighted, since the flavored oil coating introduces additional ingredients and substantially modifies the food matrix, particularly the fat content. Therefore, any differences observed between CCP and the experimental formulations cannot be attributed exclusively to the level of chickpea replacement, but they may also reflect the effects of the post-extrusion coating.

### 2.2. Sample Preparation

The snack samples were conditioned for physicochemical analyses by passing them first through a feed processor and then through a cyclone mill (UDY Corporation, model: 3010-030, Fort Collins, CO, USA), obtaining a particle size of <0.5 mm.

### 2.3. Chemical Composition Analyses

Moisture, fat, protein, ash, and dietary fiber contents of the extruded samples were determined according to official AOAC methods. Specifically, moisture, fat, protein, and ash were analyzed following AOAC methods 925.10, 945.16, and 920.87 [49] and 979.09 [50], respectively. Total dietary fiber (TDF), soluble dietary fiber (SDF), and insoluble dietary fiber (IDF) were quantified using the TDF-100A assay kit (Sigma-Aldrich, St. Louis, MO, USA) in accordance with AOAC methods 960.52 and 985.29 [51]. Phytic acid content was determined following the methodology described by Muñoz-Llandes et al. [52]. The samples were analyzed in triplicate.

The total starch content of extruded snacks was determined using the K-TSTA-100A assay kit (Megazyme Ltd., Bray, Ireland) according to AOAC Official Method 996.11 [53]. Approximately 100 mg of finely ground samples were suspended in 10 mL of sodium acetate buffer (pH 5.0) and subjected to enzymatic hydrolysis with 0.1 mL of thermostable α-amylase, followed by incubation at boiling temperature for 15 min. The resulting maltodextrins were further hydrolyzed to D-glucose by the addition of 0.1 mL of amyloglucosidase and incubation at 50 °C for 30 min. Following hydrolysis, samples were centrifuged (13,000 rpm, 5 min) and diluted (1:10). Aliquots (0.1 mL) were then mixed with 1.5 mL of glucose oxidase–peroxidase (GOPOD) reagent for glucose determination. D-glucose solutions (1 mg/mL) were used as calibration standards, and corn starch (85% starch) was included as a positive control to verify hydrolysis efficiency. Negative controls were prepared by replacing the sample with sodium acetate buffer containing CaCl_2_ (100 mM, pH 5.0) to prevent overestimation of starch hydrolysis. All standards and controls (positive and negative) were included in each assay and analyzed in parallel with the samples. Absorbance was measured at 510 nm using a UV–Vis spectrophotometer (T80, PG Instruments Ltd., Leicester, UK), and total starch content was calculated using Equation (1):

(1) $$Hidrolized\;starch\;\left(\%\right)=A\;\times F\;\times \;V_{D}\times \;\frac{D}{W_{d}}\;\times 0.9$$

where

*A* = sample absorbance;

*F* = factor to convert absorbance values to µg of D-glucose (100 µg of D-glucose divided by the GOPOD absorbance value for 100 µg of D-glucose);

*V_D_* = sample volume (mL);

*D* = dilution factor;

*W_d_* = sample dry weight (mg);

0.9 = conversion factor from free glucose (D-glucose) to anhydro D-glucose, as occurs in starch.

#### 2.3.1. Essential Amino Acids, Tyrosine and Cysteine Composition

Essential amino acids, tyrosine, and cysteine composition were determined following the method described by Lorenzo et al. [54]. Briefly, samples (100 mg) were subjected to acid hydrolysis with 5 mL of 6 N HCl in sealed glass ampoules at 110 °C for 24 h. After hydrolysis, the hydrolysates were diluted with distilled water (25 mL) and filtered through a 0.45 µm membrane filter (Filter Laboratory, Barcelona, Spain). Derivatization of samples and standards (Amino Acid Standard H, Thermo, Rockford, IL, USA) was performed using the AccQ-Fluor reagent kit (Waters Corporation, Milford, MA, USA). Aliquots (10 µL) were buffered to pH 8.8 with 70 µL of AccQ-Fluor borate buffer, followed by the addition of 20 µL of AccQ-Fluor reagent (3 mg/mL in acetonitrile). The reaction with primary and secondary amines proceeded rapidly, and excess reagent was hydrolyzed within 1 min. Samples were then heated at 55 °C for 10 min to ensure complete derivatization. Amino acid separation, identification, and quantification were carried out by high-performance liquid chromatography (HPLC) (Alliance 2695, Waters, Milford, MA, USA) equipped with a fluorescence detector (model 2475, Waters), using excitation and emission wavelengths of 250 and 395 nm, respectively. Amino acids were identified based on retention times using external standards, and results were expressed as mg/100 g protein. Tryptophan was not determined due to its degradation during acid hydrolysis.

#### 2.3.2. Mineral Profile

The mineral contents of extruded snacks (Ca, Cu, Fe, K, Mg, Mn, Na, P and Zn) were determined using inductively coupled plasma mass spectrometry (ICP-MS) model Shimadzu MS-2030 (Shimadzu, Kioto, Japan) [55]. Firstly, a 0.2 ± 0.05 g portion of the ground sample was weighed into digestion tubes. Subsequently, 8 mL of 69% nitric acid and 2 mL of hydrogen peroxide were added to each tube. The mixtures were then subjected to microwave-assisted digestion for 40 min. After digestion, the samples were diluted with Milli-Q water and brought to a final volume of 50 mL. A 10 mL aliquot of this solution was then filtered through a 0.45 μm membrane filter, and the samples were analyzed. The ICP-MS equipment operated at the following conditions: 0.91 L/min; radio frequency 1200 W, lens voltage 1.6 V; cool gas 12.0 L/min; auxiliary gas 0.70 L/min. Standard solutions of 31.25–2000 mg/L for Ca, Mg, and P and 0.78–50 mg/L for Cu, Fe, Mn, and Zn were prepared. The results were expressed in µg/g sample. All analyses were performed in triplicate.

### 2.4. Simulated In Vitro Gastrointestinal Digestion

Before the in vitro digestion simulation, the samples were crumbled and sieved in order to obtain a particle size similar to that obtained in human chewing of this type of product, using particle sizes between 1 and 4 mm. White bread was used as a reference to calculate the glycemic index. It was purchased fresh from a local bakery in Orihuela (Spain). It was a standard white bread made exclusively with refined wheat flour, water, salt, and yeast, with no added fats, sugars, or seeds that could alter the starch digestibility pattern. In this sample, a particle size of 2–4 mm was used.

The simulation of gastrointestinal digestion was followed according to the harmonized protocol of INFOGEST 2.0 [44]. The process initiated by preparing a fluid tomato consistency, mixing 1.2 g of sample (CF, F30, F60) or 1.5 g (CCP) with 4.8 mL or 3.5 mL of distilled water, respectively, in 50 mL Falcon tubes. Simulated electrolyte solutions corresponding to the oral (SSF), gastric (SGF), and intestinal (SIF) phases were prepared according to the standardized INFOGEST protocol and the concentrations established by Brodkorb et al. [44] and Minekus et al. [47] to reproduce physiological conditions.

In vitro digestion was initiated with the oral phase by adding simulated salivary fluid (SSF) at twice the initial sample volume, human salivary α-amylase (75 U/mL) (A1031-5KU, Sigma-Aldrich; 600 µL for CF, F30, F60, and 500 µL for CCP), and CaCl_2_ (0.015 M), adjusting the mixture to pH 7.00 ± 0.05. Samples were incubated at 37 °C for 2 min and 60 rpm of agitation, in a rotation mixer (ELMI Intelli-Mixer™ RM-2S; ELMI; Riga, Latvia).

The gastric phase was initiated by adding simulated gastric fluid (SGF) adjusted to pH 3.00 in order to stop oral enzymatic activity. Subsequently, pepsin (P7012, Sigma-Aldrich; 2000 U/mL) was added at volumes of 500 or 600 µL. The final pH was readjusted to 3.00 ± 0.05 using HCl (1 M and 0.5 M), and the volume was completed with distilled water to reach twice the volume of the oral phase. Samples were then incubated for 2 h under the same conditions applied during the oral phase.

Finally, the gastric digesta was mixed with simulated intestinal fluid (SIF) adjusted to pH 7.00, followed by the addition of CaCl_2_ (3 M), bovine bile extract (B3883-25G, Sigma-Aldrich; 3.0 or 2.5 mL), and pancreatin (P7545, Sigma-Aldrich, 8 × USP; 6.0 or 5.0 mL). The final pH was adjusted to 7.00 ± 0.05 using NaOH (1 M and 0.25 M), and the volume was completed with distilled water to reach twice the volume of the gastric phase. Samples were then incubated for 2 h under the same conditions applied during the previous digestion phases.

In vitro digestion was terminated by thermal shock at boiling temperature for 5 min. Subsequently, in vitro protein digestibility (IVPD) and mineral bioaccessibility (MB) were determined by collecting samples at the end of in vitro digestion (240 min, intestinal phase) for all formulations (CF, F30, F60, and CCP). Tests were performed in triplicate.

To evaluate the glycemic index, independent in vitro digestions were performed at each sampling time point: 2 min (oral phase), 10 and 120 min (gastric phase), and 140 and 240 min (intestinal phase), following the previously described protocol. All digestions were carried out in duplicate, including an additional control without enzyme addition. In total, five digestion time points were evaluated for each of the four formulations (CF, F30, F60, and CCP) and the bread reference, resulting in 15 oral, 30 gastric, and 30 intestinal digestions. After each sampling point, the reaction was stopped by thermal shock at boiling temperature for 5 min. In this test, fluid tomato consistency of samples was achieved by mixing 300 mg of snack samples (CF, F30, F60) with 1.2 mL of distilled water, whereas the same quantity of CCP sample was mixed with 0.7 mL in 15 mL Falcon tubes. The sample quantities used were optimized according to the subsequent analytical requirements and therefore differed from those employed for IVPD and mineral bioaccessibility (MB) analyses. However, the same digestion conditions and proportions established in the IVPD protocol were maintained for each phase, adjusting them to the initial sample volume.

### 2.5. In Vitro Protein Digestibility and Protein Quality Evaluation

At the end of in vitro digestion (240 min), and after stopping the reaction, digesta volumes of 24 mL (CF, F30, and F60) and 20 mL (CCP) were obtained. The supernatant was separated from the pellet by centrifugation (8400× *g*, 10 min, 25 °C; Nahita Model 2652, Alicante, Spain). The protein content in the pellet was subsequently determined using the Kjeldahl method, while the supernatant was used for mineral bioaccessibility analysis. Protein digestibility was calculated as the difference between the total protein content of each formulation and the protein quantified in the residual pellet after digestion, and expressed as the percentage of digested protein relative to the total protein content of each formulation.

#### 2.5.1. Chemical Score (CS)

The chemical score (CS) was determined by identifying the most limiting essential amino acid (EAA), based on the comparison of individual EAA contents with the reference pattern established for children and adults (≥3 years) by the Food and Agriculture Organization [56], as previously described by Salas et al. [57]. The amino acid score (AAS) was determined following the same approach as the CS, but calculated individually for each essential amino acid, as well as for cysteine and tyrosine. The CS and AAS were calculated using Equations (2) and (3):

(2) EAA content=AA (g100g)% protein×100

(3) $$CS=\left(\frac{Most\;limiting\;EAA\;content}{Recommended\;EAA\;requierement}\right)\times 100$$

#### 2.5.2. In Vitro Protein Digestibility-Corrected Amino Acid Score (IV-PDCAAS)

This index combines in vitro protein digestibility with essential amino acid composition, following the guidelines established by the Food and Agriculture Organization [56]. It was determined by multiplying the amino acid score of the limiting amino acid by the in vitro protein digestibility of each sample in Equation (4), as described by Sá et al. [58]:

(4) IV−PDCAAS=Amino acid Score×% protein digestibility

#### 2.5.3. Estimated Protein Efficiency Ratio (E-PER)

The estimated protein efficiency ratio (E-PER) predicts the ability of a protein to support growth. It was calculated from the amino acid composition of the samples using the predictive Equation (5) proposed by Alsmeyer et al. [59]:

(5) E−PER=−0.468+0.454(Leu)−0.105(Tyr)

where −0.468 is the regression intercept, whereas 0.454 and −0.105 are empirical regression coefficients corresponding to leucine and tyrosine, respectively. These coefficients were derived by Alsmeyer et al. [59] through multiple linear regression analysis to predict the protein efficiency ratio (PER) from amino acid composition and therefore do not represent the individual physiological contribution of these amino acids.

### 2.6. Bioaccessibility of Minerals After Gastrointestinal Digestion In Vitro

For this determination, 2.5 mL of the supernatant obtained at the end of in vitro protein digestion (240 min) was used. Mineral content (Ca, Cu, Fe, Mg, Mn, P, and Zn) was determined as previously described. Mineral bioaccessibility was calculated for each element using the following Equation (6):

(6) $$Mineral\;Bioaccesibility\left(\%\right)=\frac{M_{ad}}{M_{i}}\times 100$$

where

- *M_ad_:* mineral content after in vitro digestion;
- *M_i_*: initial mineral content, before in vitro digestion.

### 2.7. Kinetic of Starch Hydrolisis and Estimated Glycemic Index

Both determinations were performed following the method of Goñi et al. [60], with modifications proposed by Lucas-González et al. [61]. Starch hydrolysis kinetics were evaluated to determine the percentage of starch hydrolyzed and glucose released at each sampling time, using the same procedure described for total starch determination, except for the omission of the α-amylase addition step.

During in vitro digestion, starch was progressively hydrolyzed into maltodextrins, which were subsequently converted into D-glucose by amyloglucosidase. The amount of D-glucose released was quantified spectrophotometrically and used to determine the extent of starch hydrolysis.

After terminating digestion by thermal shock, two aliquots from digested samples and enzyme-free controls from each digestion time point (2, 10, 120, 140, and 240 min) were centrifuged (8400× *g*, 10 min, 25 °C; Nahita Model 2652, Alicante, Spain). Aliquots (0.5 mL) of the supernatant were collected in duplicate and mixed with 1.5 mL of acetate buffer (100 mM, pH 5.0), followed by the addition of 30 µL of amyloglucosidase (3300 U/mL; Megazyme Ltd., Ireland) to ensure complete hydrolysis of maltose and maltodextrins into D-glucose. The mixture was incubated at the same conditions as total starch content determination (Section 2.3).

Subsequently, appropriate dilutions were performed with acetate buffer (pH 5.0) to obtain absorbance values within the range of 0.3–0.9. Then, 100 µL aliquots were mixed with 3 mL of glucose oxidase–peroxidase (GOPOD) reagent (Megazyme Ltd., Ireland) and incubated at 50 °C for 30 min. Absorbance was measured at 510 nm using a UV–Vis spectrophotometer (T80, PG Instruments Ltd., UK). The same controls used for total starch determination were included, together with D-glucose solutions (1 mg/mL) as calibration standards in each assay.

Results were expressed as the percentage of hydrolyzed starch using Equation (1) [60]. The area under the curve (AUC) was calculated using the trapezoidal rule, which is the most robust approach to prevent underestimation in steep reaction kinetics (Equation (7)). The hydrolysis index (HI) was determined by comparing the AUC of each sample with the one coming from white bread used as a reference food (Equation (8)). Subsequently, the estimated glycemic index (eGI) was calculated from HI following Equation (9). The hydrolysis values at 240 min were considered as the final reaction extent (C∞).

(7) $$UC=\sum_{i=1}^{n-1}\left[\frac{C_{i}+C_{i+1}}{2}\times (t_{i+1}{-t}_{i})\right]$$

(8) $$HI=\frac{{AUC}_{Snack\;extruded}}{{AUC}_{white\;bread}}\times 100$$

(9) eGI=39.71+0.549HI

### 2.8. Statistical Analysis

Results were calculated as the mean ± standard deviation from three technical replicates per batch. Prior to statistical analysis, the assumptions of normality and homogeneity of variances were verified. An analysis of variance (ANOVA) was performed at 95% significance level to formulations, as the main effect, to determine the differences in chemical parameters, mineral bioaccessibility, protein quality and digestibility, starch digestibility, and estimated glycemic index (eGI). Tukey’s test was applied for the comparison of means. Statistical analyses were performed using Statgraphics Centurion 19 version software (Statgraphics Technologies, Inc., The Plains, VA, USA).

## 3. Results and Discussion

### 3.1. Chemical Composition

The chemical composition of the formulations is presented in Table 2. The highest fat content was observed in the CCP formulation (25.70 ± 0.54%), as a consequence of the post-extrusion flavored oil coating applied to this formulation (Table 1). Consequently, the higher fat content is primarily attributable to this processing step. The remaining formulations, without flavored oil coating, showed similar fat levels, ranging from 0.53 to 0.87%, in agreement with other studies [14,15,16,62].

The incorporation of the flavored oil coating in the CCP formulation resulted in an expected reduction in all other nutrients except fat and ash content. Protein content notably increased because of the chickpea incorporation, with F60 exhibiting the highest value (15.09 ± 0.19%) and CF the lowest (6.83 ± 0.34%). Similarly, ash and total dietary fiber contents increased with increasing chickpea levels, with the insoluble dietary fiber being the predominant fraction, consistent with trends reported in the literature [10,14,16,63]. Phytic acid content also increased with chickpea incorporation, ranging from 4.28 mg SPE/g sample in CF to 9.49 mg SPE/g sample in F60, following the same pattern described by Rico et al. [12]. A clear reduction in TS content was observed with increasing chickpea/rice incorporation. In this context, carbohydrate content also decreased as chickpea levels increased, at the expense of the other macronutrients, in agreement with previous findings [11,12,15,16,62,63,64].

The composition of essential amino acids, together with tyrosine and cysteine (Table 3), in combination with protein content, provides valuable insight into the nutritional quality of the formulations and the effects of chickpea incorporation and extrusion processing [65]. Overall, chickpea incorporation increased the concentration of most amino acids, primarily due to the higher protein contribution of chickpea flour. An exception was tyrosine, which was present at higher levels in the control formulation (CF).

Among the conditionally essential amino acids, cysteine increased significantly with chickpea enrichment, reaching its highest concentration in F60 (466.59 mg/100 g). This increase is likely associated with the greater protein content provided by chickpea flour, which enhanced the overall sulfur amino acid pool despite the relatively low levels of sulfur-containing amino acids typically found in legume proteins [66,67]. Consequently, the higher cysteine content may partially offset the lower methionine concentration, as both amino acids contribute to the total sulfur amino acid fraction.

In contrast, methionine exhibited a different pattern, with lower concentrations in the chickpea-enriched formulations than in CF, except F60. This observation is consistent with the well-documented limitation of legume proteins in sulfur-containing amino acids. Therefore, although chickpea supplementation markedly improved the overall amino acid profile, methionine remained comparatively low, indicating that sulfur amino acids continue to represent the primary limiting amino acids in these formulations.

Notably, chickpea incorporation substantially increased lysine content, thereby compensating for the intrinsic lysine deficiency of cereal proteins and enhancing the overall nutritional quality and amino acid balance of the formulations.

Regarding mineral composition (Table 4), chickpea/rice incorporation resulted in increased concentrations of all analyzed minerals. The higher calcium (Ca) and manganese (Mn) levels observed in the CCP formulation were attributed not only to the formulation but also to the coating formulation, which included several dairy-derived ingredients, such as milk solids, sodium caseinate, and calcium lactate and natural flavors. Specifically, Ca content reached 62.17 ± 2.69 mg/100 g in CCP, compared to 28.93 ± 4.65 mg/100 g in F60, while Mn content was 2.02 ± 0.05 mg/100 g in CCP and 1.79 ± 0.05 mg/ g in F60.

### 3.2. Protein Digestibility and Protein Quality Evaluation

**Figure 1:**
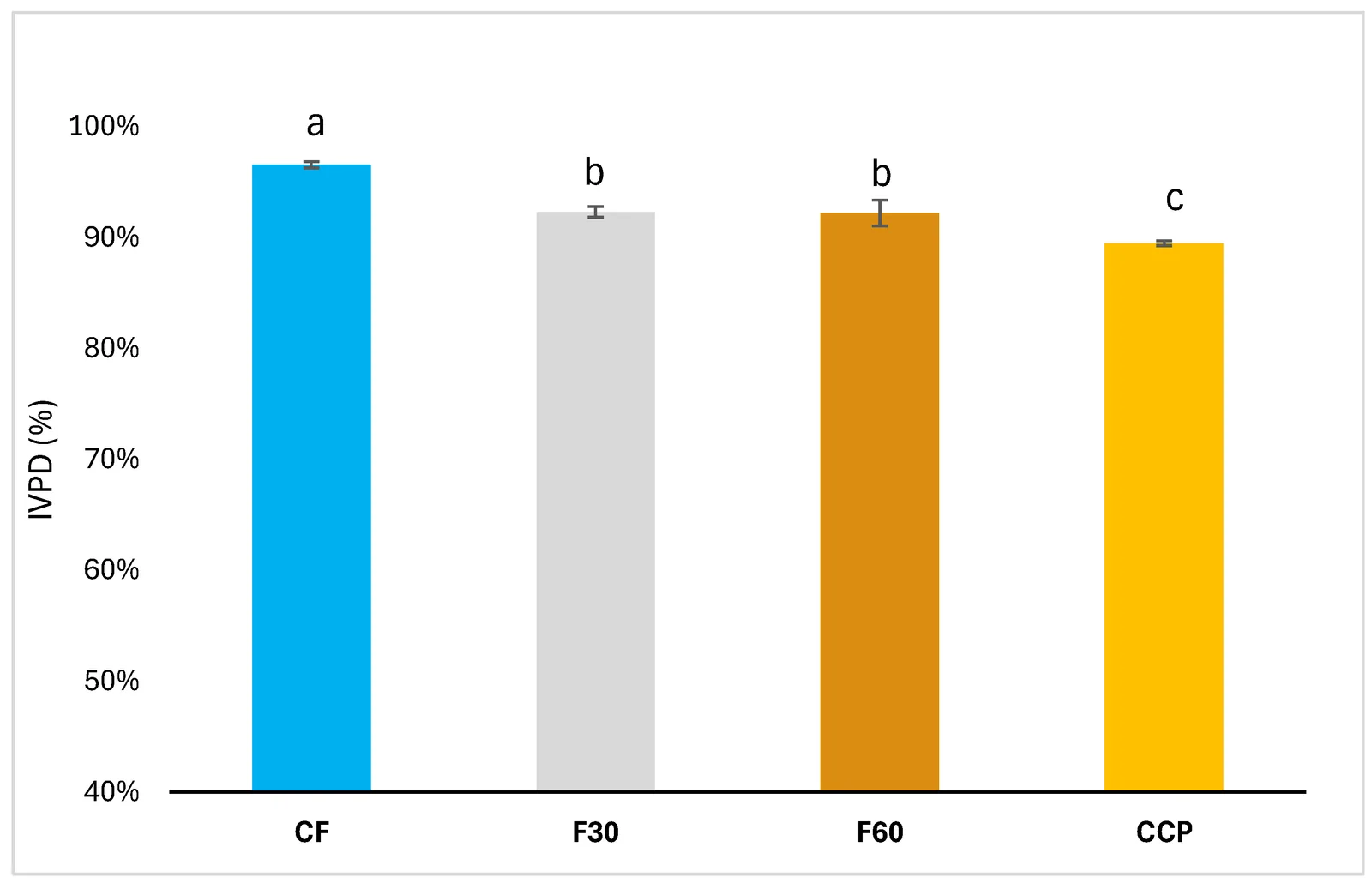
Protein digestibility of the snack formulations. Mean values (n = 3) and standard deviation. Same letters above the bars indicate no significant differences (*p* < 0.05); Tukey’s test applied. CF: control formulation 100% corn; F30: 30% chickpea, 50% rice, 20% corn; F60: 60% chickpea, 35% rice, 5% corn; CCP: commercial chickpea product 45% chickpea, 26% rice, 25% flavored oil coating, 4% corn.

Protein digestibility results are presented in Figure 1. The highest digestibility was observed in the control formulation (CF), reaching 96.56%, whereas the lowest value was recorded in CCP (89.5%). Despite its higher digestibility, CF provided the lowest amount of protein (6.60 g/100 g product), while F60 exhibited the highest value (15.09 g/100 g product). Thus, although all formulations showed high protein digestibility (89.5–96.56%), the initial protein content appears to be a more relevant determinant of the actual amount of digestible protein delivered by these products.

These values are consistent with those reported for cereal–pulse extruded products (71–95%) [16,26,63,68,69,70,71] but is in contrast to studies reporting increased digestibility with higher pulse incorporation [10,16,27].

Although the reduction in digestibility was modest, it may be attributed to the higher dietary fiber content, particularly the insoluble fraction, as well as residual antinutritional factors such as trypsin inhibitors and phytic acid [72,73]. In the present study, insoluble fiber increased from 1.38% in CF to 4.92% in F60 (Table 2), which may have limited enzymatic accessibility. In addition, phytic acid can interact with proteins via electrostatic interactions, forming complexes that reduce protein solubility and enzymatic hydrolysis [17,37,74], despite extrusion markedly reducing trypsin inhibitor activity (70–90%) and only partially decreasing phytic acid (approximately 16% reduction in chickpea) [41,75].

The lower digestibility value observed in CCP compared to F60 could be attributed to the presence of the oil coating. The higher lipid content in CCP may hinder the activity of digestive enzymes such as pepsin in the gastric phase and trypsin in the intestinal phase. Due to their hydrophilic nature, these enzymes may have limited the ability to penetrate lipid-rich matrices, thereby reducing their access to protein substrates. As a result, protein hydrolysis may be slower and less efficient, as the physical interaction between enzymes and proteins is restricted [76].

Table 5 presents the chemical score (CS) of the evaluated formulations. Lysine was identified as the limiting amino acid in CF (53%) and F30 (79.51%), whereas valine was limiting in F60 chickpea-formulation. In contrast, the CCP formulation met the essential amino acid requirements. These findings are consistent with previous studies reporting lysine as the limiting amino acid in cereal-based products [10,26,27,68,70,71,77]. Although pulses are richer in lysine, extrusion may reduce its availability due to its susceptibility to Maillard reactions [26]. While this effect was not evident in the present study, the identification of valine as the limiting amino acid agrees with previous observations in pulse-enriched extrudates [70]. From a nutritional perspective, the CCP formulation exhibited the highest protein quality according to its chemical score, fulfilling the Food and Agriculture Organization [56] indispensable amino acid reference pattern for children aged 3 years and older. F60 achieved a comparable amino acid profile, whereas CF presented the lowest chemical score and, consequently, the lowest protein quality among the evaluated formulations. These findings demonstrate the nutritional complementarity between cereal and legume proteins and support their combination as an effective strategy for improving the protein quality of novel food products.

As shown in Table 6, although CF exhibited the highest in vitro protein digestibility, it yielded the lowest in vitro protein digestibility-corrected amino acid score (IV-PDCAAS). In contrast, CCP and F60, despite showing the lowest protein digestibility, achieved the highest IV-PDCAAS values owing to their more favorable indispensable amino acid profiles. Unlike the chemical score alone, IV-PDCAAS provides a more comprehensive assessment of protein nutritional quality by integrating both indispensable amino acid composition and protein digestibility, as the latter is a key determinant of amino acid bioaccessibility. Consequently, the slightly lower digestibility of CCP was outweighed by its superior amino acid profile, resulting in the highest overall protein quality among the evaluated formulations. Similar improvements in IV-PDCAAS following chickpea incorporation were reported by Neder-Suarez et al. [27]. However, in that study the higher IV-PDCAAS reported values resulted from simultaneous improvements in both in vitro protein digestibility and chemical score.

Regarding the estimated protein efficiency ratio (E-PER), values ranged from 1.82 ± 0.04 to 5.05 ± 0.19 (Table 6). CF exhibited the highest values, whereas F60 showed the lowest, likely due to the higher leucine content per gram of protein in CF compared to chickpea-enriched formulations. This may be explained by the fact that E-PER strongly depends on the balance of specific essential amino acids, with leucine having a major contribution in this predictive equation. Therefore, the reduction in E-PER does not necessarily indicate poor protein quality but rather changes in amino acid composition resulting from chickpea/rice incorporation. Interestingly, these findings differ from those reported by Neder Suarez et al. [27], who observed an increase in E-PER following chickpea incorporation. This discrepancy may be attributed to differences in the amino acid composition of the raw materials, particularly the lower tyrosine content of the chickpea flour and the lower leucine content of the 100% maize control formulation used in their study, both of which would favor higher predicted E-PER values in the chickpea-enriched formulations.

Overall, these findings indicate that, from a nutritional perspective, the substantially higher intrinsic protein content of pulse-enriched formulations more than compensates for the slight reduction in digestibility, resulting in a net increase in the amount of digestible protein available. Moreover, the nutritional complementarity between corn and rice cereals and pulse proteins substantially improves overall protein quality by providing a more balanced indispensable amino acid profile, highlighting the value of combining these ingredients in the development of nutritionally enhanced food products.

### 3.3. Mineral Bioaccessibility

Mineral content is generally higher in pulses than in cereals such as corn, and their incorporation into extruded products can therefore improve dietary mineral intake, as previously reported [10,11,15].

Table 7 shows the mineral bioaccessibility percentages and total mineral content after digestion (I_240_). Calcium was not detected in the bioaccessible fraction of any extruded formulation analyzed. This absence may be attributed to the formation of insoluble complexes during the intestinal phase of digestion, mainly due to interactions with phytic acid, dietary fiber, phosphate species, and pH changes under alkaline conditions, which promote calcium precipitation and retention in the insoluble fraction [26,37,78]. In addition, phytic acid exhibits a preferential affinity for calcium at low concentrations, which may reduce its interaction with zinc and copper and partially explain the improved bioaccessibility patterns observed for these minerals [37,78].

As shown in Table 2, both insoluble dietary fiber and phytic acid contents increased with chickpea incorporation. Therefore, the observed reductions in mineral bioaccessibility cannot be attributed to a single factor, since both components may act simultaneously and contribute synergistically to limiting mineral release and bioaccessibility during digestion. In addition, when minerals are integrated into the food matrix, their bioavailability may be further reduced due to physical entrapment within insoluble fiber structures, which can limit their accessibility for absorption [79].

Chickpea incorporation increased copper content, whereas its bioaccessibility remained relatively low in chickpea-enriched formulations (27.82–43.22%). Notably, the control formulation (CF; 100% corn) showed no detectable copper before digestion, indicating that copper in the analyzed products primarily originated from chickpea and rice rather than corn.

In the case of iron, bioaccessibility decreased markedly from 83.18% in F30 to 71.96% in CCP and 43.87% in F60, while CF represented an exception, exhibiting the lowest iron bioaccessibility (6.97%). Overall, moderate chickpea incorporation in cereal-based formulations appeared to improve iron bioaccessibility, as observed in F30 (30% chickpea) and CCP (45% chickpea). The lower iron bioaccessibility detected in F60 compared to F30 and CCP may be associated with its higher dietary fiber and phytic acid contents, both of which have been reported to negatively affect iron bioaccessibility [80].

The results observed for zinc showed the highest bioaccessibility percentages in CF (80%) and CCP formulation (76.37%), and the lowest in F60 (44.67%). This pattern suggests a reduction in the bioaccessibility of zinc and iron, likely due to the chelating effect of phytic acid associated with chickpea incorporation. In this case, the highest zinc concentration in the supernatant after in vitro digestion for 240 min was observed in F30 (1.75 µg/g), indicating that insoluble dietary fiber and phytic acid exerted a pronounced effect in reducing zinc bioaccessibility and, consequently, its potential utilization by the organism. Previous in vivo studies have shown that phytic acid has a greater negative effect on zinc absorption than dietary fiber (soluble and insoluble), although both compounds may reduce mineral bioavailability, mainly at the duodenal level [36].

Compared with previous studies on pulse-enriched extruded products, the formulations analyzed in the present study showed higher iron and zinc bioaccessibility values than those reported by Pastor-Cavada et al. [71], likely due to differences in formulation, extrusion conditions, and matrix interactions during digestion [37,80]. In addition, moderate chickpea incorporation favored iron bioaccessibility, whereas higher chickpea levels reduced zinc bioaccessibility.

Magnesium content increased with chickpea incorporation, although no significant differences were observed in its bioaccessibility. However, magnesium concentration in the supernatant after 240 min of in vitro digestion increased markedly with chickpea incorporation, reaching 65.17 µg/g in F60 compared to 14.91 µg/g in CF.

Manganese bioaccessibility increased with chickpea content, with the highest manganese bioaccessibility (MB) observed in CCP (51.02%) and the lowest in CF (15.94%). The higher initial manganese levels detected in CCP may be attributed to the additional contribution of this mineral from the flavored oil coating. Furthermore, manganese derived from the flavored oil coating may be more bioaccessible because it is not embedded within the food matrix [79], which could explain the greater manganese bioaccessibility observed in CCP. In the case of magnesium, it has been reported that the presence of fermentable carbohydrates (e.g., fructooligosaccharides and resistant starch) can enhance its absorption. Moreover, in magnesium-rich foods such as pulses, the high intrinsic mineral content may outweigh the inhibitory effects of antinutritional factors, including dietary fiber, which can otherwise limit mineral uptake [40]. This behavior was also observed in the present study, where no significant differences in magnesium bioaccessibility were detected among formulations, despite the higher dietary fiber content associated with chickpea incorporation.

The bioaccessible phosphorus content after in vitro digestion was higher in chickpea-enriched samples, reaching the highest levels in F60 (165.2 µg/g) and the lowest in CF (61.5 µg/g). However, no clear trend was observed between phosphorus bioaccessibility (%) and chickpea content, as CF exhibited the highest bioaccessibility (88.14%) and F30 the lowest (56.53%). Therefore, no consistent effect of insoluble dietary fiber and phytic acid on phosphorus bioaccessibility was identified. Overall, the higher initial phosphorus content provided by chickpea largely compensated for the lower bioaccessibility observed in chickpea-enriched formulations, resulting in greater amounts of bioaccessible phosphorus in these samples.

Overall, although mineral bioaccessibility (% MB) varied depending on the element, higher chickpea incorporation generally resulted in increased mineral concentrations in the supernatant after 240 min of in vitro digestion, except for calcium.

### 3.4. Kinetic of Starch Hydrolysis and Estimated Glycemic Index

**Figure 2:**
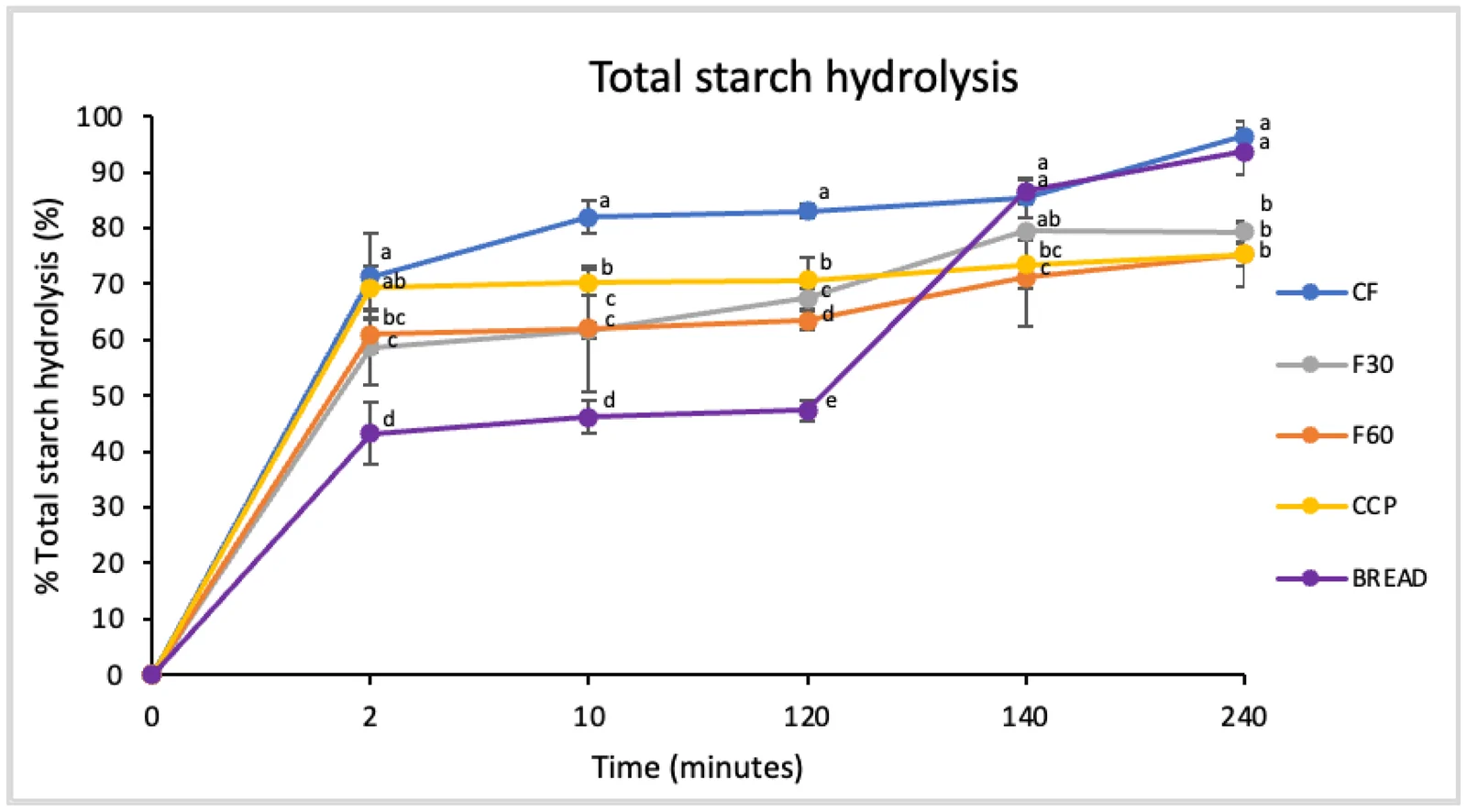
Total hydrolysis rate of the snack formulations during gastrointestinal digestion in vitro (INFOGEST 2.0 protocol). Values at the same time with the same letter do not differ significantly (*p* < 0.05); Tukey’s test applied. CF: control formulation 100% corn; F30: 30% chickpea, 50% rice, 20% corn; F60: 60% chickpea, 35% rice, 5% corn; CCP: commercial chickpea product 45% chickpea, 26% rice, 25% flavored oil coating, 4% corn.

The starch hydrolysis profiles of the analyzed formulations are shown in Figure 2. Most starch hydrolysis of the extruded snacks (60.95% to 71.35%) occurred during the oral phase by the action of salivary α-amylase, specifically within the first 2 min of digestion. In contrast, the intestinal phase, mediated by pancreatic α-amylase, was responsible for less than 15% of the total hydrolysis in FC, F30, and F60, and was practically residual (<5%) in the case of CCP, where the hydrolysis plateau was essentially reached immediately after the oral digestion step. Furthermore, the concentration on the equilibrium (Table 8) was significantly higher in CF than the other chickpea extruded formulations, in which incomplete starch hydrolysis was reported (~79%) at the end of the in vitro gastrointestinal process. These results are consistent with previous findings on extruded cereals. Zhao et al. [81] recently reported that extruded rice, wheat, and corn achieved approximately 85% starch hydrolysis before 15 min of digestion, reaching a plateau shortly thereafter. Moreover, the authors compared the starch hydrolysis of extruded cereals with their non-extruded counterparts and reported a greatly negative impact of extrusion on starch digestion, which promoted a significant increase in starch digestibility across the three studied cereals [81].

This particular kinetic pattern—characterized by an extreme initial burst followed by a rapid plateau—precluded the application of a conventional first-order kinetic model (R^2^ < 0.4). Forcing a linear adjustment across the entire 140 min timeframe to obtain a single kinetic constant (k) would have severely masked the true ultra-rapid digestibility of these samples. Consequently, to precisely quantify the actual extent of enzymatic hydrolysis without imposing inadequate kinetic assumptions, we adopted the model-independent trapezoidal rule to calculate the area under the curve (AUC). This approach provides reliable hydrolysis index (HI) and estimated glycemic index (eGI) values, which are summarized together with the total starch (TS) and total starch hydrolyzed at 140 min in Table 8.

As previously mentioned, total starch content varied significantly among formulations (*p* < 0.05), ranging from 72.0% (CF) to 48.3% (CCP) (Table 2). The progressive reduction observed in F30 (69.6%) and F60 (57.2%) compared to CF reflects the dilution effect caused by the incorporation of chickpea, despite the presence of rice in those formulations with higher starch contents than corn [23,82]. Interestingly, the absolute starch hydrolyzed at 140 min and the AUC did not strictly follow the TS trend. While CF presented the highest AUC, the CCP formulation, despite having the lowest TS content (48.3%), displayed a remarkably high AUC, which was statistically comparable to CF (*p* > 0.05). This indicates that starch quality and matrix fragility outweigh total starch quantity in determining the extent of hydrolysis. In contrast, F30 and F60 showed significantly lower AUC values (*p* < 0.05), indicating that the inclusion of chickpea effectively protected a proportion of the starch from enzymatic attack, likely through increased viscosity, fiber–starch interactions, or a more compact extruded microstructure.

The HI confirmed the ultra-digestible nature of all extruded formulations. CF exhibited the highest values (*p* < 0.05), indicating a digestibility significantly superior to that of white bread (reference, HI = 100). These results align with reported values reported for highly expanded cereals, where the extrusion process completely destroyed the microstructure of the starch granule. It basically modified the crystalline and ordered structure of native starch granules into amorphous flakes and incomplete starch granules, leaving no physical barriers to enzymatic action [81,83]. The addition of chickpea in F30 and F60 successfully attenuated this response, significatively reducing its HI. The results of the present study are consistent with Hardacre et al. [64], who demonstrated that cereal–legume combinations can reduce starch hydrolysis compared to cereal-only or pulse-only extrudates. In contrast, Nongmaithem et al. [84] reported higher starch hydrolysis values (87.61–92.5%) in corn-based formulations containing 20% germinated lentil, exceeding those obtained in this study for chickpea-based formulations (75.3 ± 5.78 and 79.25 ± 1.92), except for CF, which showed the highest value (96.4 ± 2.90%) in minute 240.

The observed starch hydrolysis behavior is directly reflected in the glycemic response of the products. The glycemic index (GI) is defined as the postprandial blood glucose response to 50 g of available carbohydrates from a test food relative to a reference food (glucose or white bread) [85]. It is a key nutritional parameter used to classify foods based on their glycemic and insulin responses, which are associated with fat storage and adiposity-related weight gain [86]. The highest values were observed for CF and CCP, while the lowest values corresponded to the formulation with the highest chickpea percentage (F60) (Table 8). Although the eGI values of F30 and F60 remain above 100, the significant reduction (*p* < 0.05) compared to CF confirms that the formulation strategy effectively lowers the glycemic potential.

A comparison between our in vitro findings and the published in vivo literature shows strong consistency, with values observed up to 100. For instance, Foster-Powell et al. [87] reported glycemic index values of 106 for corn- and rice-based extruded products, while Johnston et al. [14] reported an in vivo glycemic response of 110 mmol·min/L (0–120 min) for formulations containing 40% chickpea, both higher than those observed in the present study.

Although the reduction in eGI for CCP and F60 was modest relative to CF, the higher dietary fiber content (predominantly insoluble fiber derived from chickpea), as well as the potential presence of resistant starch (both intrinsic to chickpea and formed during extrusion, i.e., type 3 resistant starch), may have limited α-amylase activity during in vitro digestion [17,88,89,90,91].

In summary, chickpea incorporation contributed to a partial reduction in starch hydrolysis and eGI, likely as a result of both the lower total starch content and the increased resistant starch fraction in the enriched formulations. Accordingly, F60 and CCP exhibited the most favorable results. However, eGI values remained high across all formulations when compared with the reference bread, highlighting the need for additional strategies to further reduce glycemic response, as well as validation through in vivo studies to minimize potential overestimation.

## 4. Conclusions

The partial replacement of corn by chickpea/rice combinations in extruded snacks significantly improved their nutritional profile by increasing protein, dietary fiber, and mineral contents. Although the protein digestibility and mineral bioaccessibility of phosphorus, magnesium and zinc were slightly reduced in chickpea/rice-enriched formulations, these effects were offset by the substantially higher initial nutrient content of protein and minerals. In fact, the commercial chickpea product showed the highest protein digestibility-corrected amino acid score despite the lower protein digestibility. In terms of starch digestion, chickpea incorporation moderated starch hydrolysis compared to the 100% corn formulation, likely due to its lower starch content and matrix effects associated with dietary fiber and protein. However, starch hydrolysis and estimated glycemic index (eGI) values remained high relative to the reference food (bread), indicating the need for further optimization. Overall, these findings’ in vitro tests demonstrate that chickpea/rice substitution represents an effective strategy to improve the nutritional quality of extruded snacks. However, these promising results should be confirmed by the application of in vivo tests to evaluate the direct health benefits.

## Figures and Tables

**Table 1:** Ingredient composition of snack formulations.

Raw Materials	Formulations			
	CF	F30	F60	CCP
Chickpea (%)	-	30	60	45
Rice (%)	-	50	35	26
Corn (%)	100	20	5	4
Flavored oil coating (%)	-	-	-	25
TOTAL	100	100	100	100

**Table 2:** Chemical parameters of different snack formulations.

Chemical Parameters	Formulations			
	CF	F30	F60	CCP
Moisture (%)	6.62 ± 0.11 ^a^	5.53 ± 0.10 ^c^	5.85 ± 0.10 ^b^	5.05 ± 0.19 ^d^
Fat (%)	0.78 ± 0.10 ^b^	0.53 ± 0.16 ^b^	0.87 ± 0.12 ^b^	25.70 ± 0.54 ^a^
Protein (%)	6.83 ± 0.34 ^c^	10.41 ± 0.05 ^b^	15.09 ± 0.19 ^a^	10.48 ± 0.08 ^b^
Ash (%)	0.17 ± 0.01 ^d^	1.35 ± 0.01 ^c^	2.04 ± 0.03 ^b^	3.31 ± 0.08 ^a^
Total dietary fiber, TDF (%)	2.45 ± 0.01 ^a^	3.34 ± 0.88 ^a^	5.12 ± 1.58 ^a^	3.71 ± 0.67 ^a^
Soluble dietary fiber, SDF (%)	1.08 ± 0.00 ^a^	0.98 ± 0.38 ^a^	1.08 ± 0.13 ^a^	0.78 ± 0.09 ^a^
Insoluble dietary fiber, IDF (%)	1.38 ± 0.02 ^c^	2.81 ± 0.78 ^b^	4.92 ± 0.56 ^a^	3.11 ± 0.11 ^b^
Carbohydrate (%)	83.40 ± 0.08 ^a^	78.84 ± 0.78 ^b^	71.03± 1.49 ^c^	51.68 ± 0.81 ^d^
Total starch (%)	72.02 ± 1.65 ^a^	69.62 ± 1.97 ^b^	57.22 ± 2.33 ^c^	48.29 ± 1.19 ^d^
Phytic acid (mgSPE */g of sample)	4.28 ± 0.39 ^d^	8.82 ± 0.47 ^b^	9.49± 0.13 ^a^	7.15 ± 0.11 ^c^

Mean values (n = 3), standard deviation. Values in the same row with the same letter do not differ significantly (*p* > 0.05). * Sodium Phytate Equivalents (SPE). CF: control formulation 100% corn; F30: 30% chickpea, 50% rice, 20% corn; F60: 60% chickpea, 35% rice, 5% corn; CCP: commercial chickpea product 45% chickpea, 26% rice, 25% flavored oil coating, 4% corn.

**Table 3:** Essential amino acids, tyrosine and cysteine composition of different snack formulations.

Essential Amino Acids, Tyrosine and Cysteine Composition (mg/100 g Sample)	Formulations			
	CF	F30	F60	CCP
Hys *	207.80 ± 1.66 ^d^	252.32 ± 6.94 ^c^	440.75 ± 5.72 ^a^	319.03 ± 4.14 ^b^
Thr *	296.51 ± 18.65 ^d^	368.54 ± 18.32 ^c^	614.18 ± 30.28 ^a^	444.57 ± 21.92 ^b^
Tyr **	281.70 ± 0.39 ^a^	113.08 ± 3.12 ^d^	178.21 ± 6.62 ^b^	128.99 ± 4.79 ^c^
Cys **	310.83 ± 21.55 ^c^	374.90 ± 29.53 ^b^	466.59 ± 6.58 ^a^	337.73 ± 4.77 ^c^
Val *	387.80 ± 26.32 ^d^	453.58 ± 28.72 ^c^	688.09 ± 19.92 ^a^	498.06 ± 14.42 ^b^
Met *	67.98 ± 2.99 ^b^	57.25 ± 3.20 ^c^	79.43 ± 1.48 ^a^	57.49 ± 1.07 ^c^
Lys *	179.09 ± 2.24 ^d^	476.29 ± 21.85 ^c^	928.59 ± 12.33 ^a^	672.15 ± 8.93 ^b^
Ile *	286.49 ± 12.12 ^d^	376.31 ± 12.11 ^c^	671.35 ± 16.47 ^a^	485.95 ± 11.92 ^b^
Leu *	1013.67 ± 29.40 ^b^	784.52 ± 5.98 ^d^	1155.22 ± 7.21 ^a^	836.19 ± 5.22 ^c^
Phe *	393.88 ± 0.85 ^d^	483.95 ± 43.26 ^c^	920.34 ± 26.96 ^a^	666.17 ± 19.52 ^b^

Dry basis. Mean values (n = 3), standard deviation. Values in the same row with the same letter do not differ significantly (*p* > 0.05). * Essential amino acids; ** essential amino acids under certain conditions. CF: control formulation 100% corn; F30: 30% chickpea, 50% rice, 20% corn; F60: 60% chickpea, 35% rice, 5% corn; CCP: commercial chickpea product 45% chickpea, 26% rice, 25% flavored oil coating, 4% corn.

**Table 4:** Mineral content of the different snack formulations.

Formulations	CF	F30	F60	CCP
Ca (mg/100 g)	7.19 ± 2.39 ^d^	18.58 ± 2.43 ^c^	28.93 ± 4.65 ^b^	62.17 ± 2.69 ^a^
Cu (mg/100 g)	nd	0.22 ± 0.02 ^b^	0.37 ± 0.02 ^a^	0.26 ± 0.03 ^b^
Fe (mg/100 g)	1.17 ± 0.06 ^d^	1.72 ± 0.23 ^c^	2.84 ± 0.14 ^a^	2.52 ± 0.13 ^b^
Mg (mg/100 g)	19.66 ± 1.01 ^d^	64.56 ± 1.61 ^c^	94.86 ± 0.54 ^a^	73.91 ± 1.19 ^b^
Mn (mg/100 g)	0.13 ± 0 ^d^	1.41 ± 0.05 ^c^	1.79 ± 0.05 ^b^	2.02 ± 0.05 ^a^
P (mg/100 g)	63.27 ± 2.01 ^c^	185.77 ± 10.58 ^b^	256.94 ± 3.06 ^a^	199.45 ± 12.78 ^b^
Zn (mg/100 g)	0.86 ± 0.22 ^c^	1.75 ± 0.05 ^b^	2.53 ± 0.18 ^a^	1.85 ± 0.17 ^b^

Mean values (n = 3), standard deviation. Values in the same row with the same letter do not differ significantly (*p* > 0.05). nd: not detected. CF: control formulation 100% corn; F30: 30% chickpea, 50% rice, 20% corn; F60: 60% chickpea, 35% rice, 5% corn; CCP: commercial chickpea product 45% chickpea, 26% rice, 25% flavored oil coating, 4% corn.

**Table 5:** Protein value, limiting amino acids and protein score of the snack formulations.

Protein Value, Limiting Amino Acids and Protein Score		Formulations				EAA Requirements (3 Years and Older) * (g/100 g Protein)
		CF	F30	F60	CCP	
Hys	EAA (g/100 g protein)	2.94 ± 0.02 ^a^	2.02 ± 0.06 ^d^	2.47 ± 0.03 ^c^	2.59 ± 0.03 ^b^	1.60
	AAS (%)	184.04 ± 1.47 ^a^	126.37 ± 3.47 ^d^	154.19 ± 2.00 ^c^	161.68 ± 2.10 ^b^	
Ile	EAA (g/100 g protein)	4.06 ± 0.17 ^a^	3.02 ± 0.10 ^c^	3.76 ± 0.09 ^b^	3.94 ± 0.10 ^ab^	3.00
	AAS (%)	135.32 ± 5.73 ^a^	101.51 ± 3.24 ^c^	125.26 ± 3.07 ^b^	131.35 ± 3.22 ^ab^	
Leu	EAA (g/100 g protein)	14.36 ± 0.42 ^a^	6.29 ± 0.05 ^c^	6.47 ± 0.04 ^bc^	6.78 ± 0.04 ^b^	6.10
	AAS (%)	235.48 ± 6.83 ^a^	103.05 ± 0.78 ^c^	106.00 ± 0.66 ^bc^	111.15 ± 0.69 ^b^	
Lys	EAA (g/100 g protein)	2.54 ± 0.03 ^d^	3.82 ± 0.18 ^c^	5.20 ± 0.07 ^b^	5.45 ± 0.07 ^a^	4.80
	AAS (%)	52.87 ± 0.66 ^d^	79.51 ± 3.65 ^c^	108.29 ± 1.44 ^b^	113.55 ± 1.51 ^a^	
Met + Cys	EAA (g/100 g protein)	5.37 ± 0.35 ^a^	3.46 ± 0.26 ^b^	3.06 ± 0.05 ^b^	3.20 ± 0.05 ^b^	2.30
	AAS (%)	233.39 ± 15.12 ^a^	150.55 ± 11.40 ^b^	132.88 ± 1.96 ^b^	139.34 ± 2.06 ^b^	
Phe + Tyr	EAA (g/100 g protein)	9.57 ± 0.02 ^a^	4.78 ± 0.37 ^c^	6.15 ± 0.19 ^b^	6.45 ± 0.20 ^b^	4.10
	AAS (%)	233.49 ± 0.43 ^a^	116.68 ± 9.06 ^c^	149.98 ± 4.58 ^b^	157.26 ± 4.81 ^b^	
Thr	EAA (g/100 g protein)	4.20 ± 0.26 ^a^	2.95 ± 0.15 ^c^	3.44 ± 0.17 ^b^	3.60 ±0.18 ^b^	2.50
	AAS (%)	168.07 ± 10.57 ^a^	118.12 ± 5.87 ^c^	137.51 ± 6.78 ^b^	144.19 ± 7.11 ^b^	
Val	EAA (g/100 g protein)	5.50 ± 0.37 ^a^	3.63 ± 0.23 ^b^	3.85 ± 0.11 ^b^	4.04 ± 0.12 ^b^	4.00
	AAS (%)	137.38 ± 9.33 ^a^	90.86 ± 5.75 ^b^	96.29 ± 2.79 ^b^	100.96 ± 2.92 ^b^	
Limiting Amino Acid		Lys	Lys	Val	-	
Chemical Score (%)		52.87 ± 0.66 ^c^	79.51 ± 3.65 ^a^	96.29 ± 2.79 ^b^	100 ± 0.00 ^a^	

Mean values (n = 3) and standard deviation. Values in the same row with the same letter do not differ significantly (*p* > 0.05). AAS: amino acid score, EAA: essential amino acid. * amino acid requirement for children 3 and older according to FAO [56]. CF: control formulation 100% corn; F30: 30% chickpea, 50% rice, 20% corn; F60: 60% chickpea, 35% rice, 5% corn; CCP: commercial chickpea product 45% chickpea, 26% rice, 25% flavored oil coating, 4% corn.

**Table 6:** Protein quality parameters of the snack formulations.

Protein Quality	Formulations			
	CF	F30	F60	CCP
IV-PDCAAS	51.05 ± 0.73 ^c^	73.38 ± 2.99 ^b^	88.78 ± 2.10 ^a^	89.49 ± 0.71 ^a^
E-PER	5.05 ± 0.19 ^a^	1.88 ± 0.02 ^b^	1.82 ± 0.04 ^b^	1.93 ± 0.04 ^b^

Mean values (n = 3) and standard deviation. Values in the same row with the same letter do not differ significantly (*p* < 0.05); Tukey’s test applied. IV-PDCAAS: in vitro protein digestibility-corrected amino acid score, E-PER: estimated protein efficiency ratio. CF: control formulation 100% corn; F30: 30% chickpea, 50% rice, 20% corn; F60: 60% chickpea, 35% rice, 5% corn; CCP: commercial chickpea product 45% chickpea, 26% rice, 25%, flavored oil coating, 4% corn.

**Table 7:** Mineral content and bioaccessibility of the snack formulations.

Mineral Content		Formulations			
		CF	F30	F60	CCP
Ca	I_240_ (mg/100 g)	nd	nd	nd	nd
	% MB	nd	nd	nd	nd
Cu	I_240_ (mg/100 g)	nd	0.10 ± 0.00 ^b^	0.14 ± 0.01 ^a^	0.07 ± 0.02 ^c^
	% MB	nd	43.22 ± 3.02 ^a^	36.06 ± 2.68 ^ab^	27.82 ± 10.96 ^b^
Fe	I_240_ (mg/100 g)	0.08 ± 0.03 ^b^	1.09 ± 0.50 ^a^	1.26 ± 0.43 ^a^	1.81 ± 0.14 ^a^
	% MB	6.97 ± 2.81 ^b^	83.18 ± 13.82 ^a^	43.87 ± 12.75 ^ab^	71.96 ± 6.68 ^a^
Mg	I_240_ (mg/100 g)	14.91 ± 2.11 ^c^	43.04 ± 2.40 ^d^	65.17 ± 11.60 ^a^	53.83 ± 3.31 ^ab^
	% MB	76.24 ± 13.64 ^a^	66.68 ± 3.68 ^a^	68.66 ± 11.84 ^a^	72.88 ± 5.51 ^a^
Mn	I_240_ (mg/100 g)	0.02 ± 0.00 ^d^	0.23 ± 0.03 ^c^	0.42 ± 0.03 ^b^	1.03 ± 0.01 ^a^
	% MB	15.94 ± 1.11 ^c^	16.24 ± 1.89 ^c^	23.47 ± 2.41 ^b^	51.02 ± 5.23 ^a^
P	I_240_ (mg/100 g)	61.50 ± 19.09 ^b^	105.00 ± 12.20 ^b^	165.20 ± 39.72 ^a^	155.30 ± 7.22 ^a^
	% MB	88.14 ± 15.39 ^a^	56.53 ± 5.79 ^c^	64.18 ± 14.77 ^bc^	77.94 ± 2.21 ^ab^
Zn	I_240_ (mg/100 g)	0.67 ± 0.04 ^c^	1.71 ± 0.49 ^a^	1.13 ± 0.04 ^bc^	1.41 ± 0.11 ^ab^
	% MB	80.00 ± 16.16 ^ab^	68.03 ± 3.90 ^a^	44.67 ± 3.64 ^b^	76.37 ± 3.29 ^ab^

Mean values (n = 3) and standard deviation. Values in the same row with the same next letter do not differ significantly (*p* < 0.05); Tukey’s test applied. nd: not detected. CF: control formulation 100% corn; F30: 30% chickpea, 50% rice, 20% corn; F60: 60% chickpea, 35% rice, 5% corn; CCP: commercial chickpea product 45% chickpea, 26% rice, 25% flavored oil coating, 4% corn; I_240_: soluble mineral content after 240 min. of in vitro digestion; MB: mineral bioaccessibility.

**Table 8:** Total starch hydrolyzed at 140 and 240 min (SH140 and SH240), area under curve (AUC), hydrolysis index (HI), and estimated index glycemic (eGI) of the snack formulations at 140 min.

	SH_240_	SH_140_	AUC_140_	HI_140_	eGI_140_
CF	96.4 ± 3.2 ^a^	85.4 ± 3.6 ^a^	10,888 ± 874 ^a^	166.1 ± 1.7 ^a^	130.9 ± 1.0 ^a^
F30	79.5 ± 1.4 ^b^	79.5 ± 0.3 ^ab^	9161 ± 77 ^b^	132.9 ± 1.1 ^bc^	112.7 ± 0.6 ^bc^
F60	75.4 ± 2.0 ^b^	71.1 ± 8.7 ^b^	8855 ± 626 ^b^	128.5 ± 9.1 ^c^	110.2 ± 5.0 ^c^
CCP	75.3 ± 1.5 ^b^	73.5 ± 4.3 ^b^	9894 ± 174 ^ab^	143.5 ± 2.5 ^b^	118.5 ± 1.4 ^b^

Mean values (n = 3) and standard deviation. Values in the same column with the same letter do not differ significantly (*p* < 0.05); Tukey’s test applied; CF: control formulation 100% corn; F30: 30% chickpea, 50% rice, 20% corn; F60: 60% chickpea, 35% rice, 5% corn; CCP: commercial chickpea product 45% chickpea, 26% rice, 25% flavored oil coating, 4% corn.

## Data Availability

Dataset available on request from the authors.
